# Molecular expression, characterization and mechanism of ALAS2 gain-of-function mutants

**DOI:** 10.1186/s10020-019-0070-9

**Published:** 2019-01-24

**Authors:** Vassili Tchaikovskii, Robert J. Desnick, David F. Bishop

**Affiliations:** Department of Genetics and Genomic Sciences, Icahn School of Medicine at Mount Sinai New York, New York, 10029 USA

**Keywords:** X-linked sideroblastic anemia, Porphyria, Erythropoietic, Polymorphism, Single nucleotide, Glycine; succinyl-coenzyme a

## Abstract

**Background:**

X-linked protoporphyria (XLP) (MIM 300752) is an erythropoietic porphyria due to gain-of-function mutations in the last exon (Ducamp et al., Hum Mol Genet 22:1280-88, 2013) of the erythroid-specific aminolevulinate synthase gene (ALAS2). Five ALAS2 exon 11 variants identified by the NHBLI Exome sequencing project (p.R559H, p.E565D, p.R572C, p.S573F and p.Y586F) were expressed, purified and characterized in order to assess their possible contribution to XLP. To further characterize the XLP gain-of-function region, five novel ALAS2 truncation mutations (p.P561X, p.V562X, p.H563X, p.E569X and p.F575X) were also expressed and studied.

**Methods:**

Site-directed mutagenesis was used to generate ALAS2 mutant clones and all were prokaryotically expressed, purified to near homogeneity and characterized by protein and enzyme kinetic assays. Standard deviations were calculated for 3 or more assay replicates.

**Results:**

The five ALAS2 single nucleotide variants had from 1.3- to 1.9-fold increases in succinyl-CoA V_max_ and 2- to 3-fold increases in thermostability suggesting that most could be gain-of-function modifiers of porphyria instead of causes. One SNP (p.R559H) had markedly low purification yield indicating enzyme instability as the likely cause for XLSA in an elderly patient with x-linked sideroblastic anemia. The five novel ALAS2 truncation mutations had increased V_max_ values for both succinyl-CoA and glycine substrates (1.4 to 5.6-fold over wild-type), while the K_m_s for both substrates were only modestly changed. Of interest, the thermostabilities of the truncated ALAS2 mutants were significantly lower than wild-type, with an inverse relationship to V_max_ fold-increase.

**Conclusions:**

Patients with porphyrias should always be assessed for the presence of the ALAS2 gain-of-function modifier variants identified here. A key region of the ALAS2 carboxyterminal region is identified by the truncation mutations studied here and the correlation of increased thermolability with activity suggests that increased molecular flexibility/active site openness is the mechanism of enhanced function of mutations in this region providing further insights into the role of the carboxyl-terminal region of ALAS2 in the regulation of erythroid heme synthesis.

**Electronic supplementary material:**

The online version of this article (10.1186/s10020-019-0070-9) contains supplementary material, which is available to authorized users.

## Introduction

Erythroid-specific 5-aminolevulinate synthase (ALAS2, EC 2.3.1.37), is the first and rate-limiting enzyme in the erythroid heme biosynthetic pathway It is encoded by an X-linked gene (Bishop et al. 1990) and is expressed in fetal liver and adult bone marrow to provide sufficient heme for hemoglobin synthesis. ALAS2 uses pyridoxal-5′ phosphate to catalyze the condensation of glycine and succinyl-CoA to produce 5-aminolevulinic acid (ALA), the first heme precursor. X-Linked Sideroblastic Anemia (XLSA) is caused by over 80 mutations in ALAS2 (Cotter et al. 1992; Stenson et al. 2017), with nearly all located in exons 5 through 11. Recently, attention has been focused on mutations in the 33-residue carboxyl-terminal exon 11, since these mutations can cause XLSA without decreasing the in vitro catalytic activity (Bishop et al. 2012) or, more typically, cause X-Linked Protoporphyria (XLP) via overproduction of ALA (Whatley et al. 2008) and subsequent heme biosynthetic intermediates without causing anemia. These XLP gain-of-function mutations result in extreme photosensitivity due to protoporphyrin IX accumulation and the development of severe phototoxic reactions upon exposure to sunlight. The divergent and unique consequences of ALAS2 exon 11 mutations suggested that this exon serves as an intrinsic regulator of ALA synthesis (Kadirvel et al. 2012; Fratz et al. 2015). This 3′ carboxyl-terminal extension encodes a well-conserved amino acid region in higher eukaryotes, but is not present in bacteria, the source of the first crystal structure of ALAS (Astner et al. 2005). Recently, the yeast ALAS 3D structure was determined providing the first insights into the location of the carboxyl-terminal region partially homologous to human exon 11 (Brown et al. 2018).

Previously, we demonstrated that purified recombinant human ALAS2 enzymes from XLP patients with mutations in the gain-of-function region led to 1.6- to 3.1-fold increased enzymatic specific activity (Bishop et al. 2013). With others, we established the gain-of-function region to be between ALAS2 residues 533–580 (Bishop et al. 2013; Ducamp et al. 2013).

To assess whether other known exon 11 missense mutations (identified as polymorphisms in the NHLBI Exome Sequencing Project [ESP] Exome Variant Server database of 13,006 chromosomes [release ESP6500]) (Exome Variant Server 2017) may cause XLSA or XLP, five novel missense mutations were identified as being the only significant enzyme variants in ALAS2 exon 11. These variants were introduced into human ALAS2 expression constructs, overexpressed, purified, and characterized. To further characterize this region, five truncation mutations were also generated and characterized to help fine-map the region of greatest gain-of-function.

## Materials and methods

The reagents used, preparation of ALAS2 expression constructs, and the expression and purification of ALAS2 recombinant enzymes were as previously described (Bishop et al. 2013). Positive clones for missense mutations (p.R559H, p.E565D, p.R572C, p.S573F, & p.Y586F) were identified by restriction analysis and confirmed by subsequent sequence analyses. Truncated constructs (p.P561X, p.V562X, p.H563X, p.E569X and p.F575X) were made by deleting appropriate coding nucleotides of ALAS2 exon 11 using primers that included stop codons TAG or TAA as shown in Additional file 1: Table S1.

Enzyme protein concentrations, kinetics, and thermostability studies were conducted using endpoint assays as previously described (Bishop et al. 2013). Note that our choice of 45 °C as the denaturation temperature for stability studies was chosen to maximize discrimination between structural forms. Oligonucleotide primers used for mutagenesis and sequence analyses are listed in Additional file 1 Table S1.

## Results

### Expression and purification of wild-type and mutant ALAS2 enzymes

Each of the five human ALAS2 missense mutations (p.R559H, p.E565D, p.R572C, p.S573F, and p.Y586F) and five ALAS2 truncation mutations (p.P561X, p.V562X, p.H563X, p.E569X and p.F575X) were prokaryotically overexpressed. They were purified to near homogeneity in three steps with a 15–40% yield; resulting 2–8 mg of enzyme per liter of cells (Table 1; purification data for p.E569X is not shown).

**Table 1:** Purification of recombinant wild-type and mutant ALAS2^a^

ALAS2 enzyme	Step	Activity	Specific.activity	Yield	Fold Purification
		Units	Units/mg	%	fold
Wild type	Crude extract	571,000	1130	100	1
	Affinity chromatography	323,000	21,500	56	20
	Gel filtration	131,000	93,500	23	83
p.Arg559His	Crude Extract	1,090,000	2090	100	1
	Affinity chromatography	358,000	29,600	33	14
	Gel filtration	95,600	108,000	8.8	52
p.Glu565Asp	Crude extract	1,030,000	1710	100	1
	Affinity chromatography	825,000	50,200	80	29
	Gel filtration	194,000	122,000	19	71
p.Arg572Cys	Crude extract	267,000	780	100	1
	Affinity chromatography	143,000	8760	53	11
	Gel filtration	43,500	121,000	16	155
p.Ser573Phe	Crude extract	666,000	1070	100	1
	Affinity chromatography	301,000	11,400	45	11
	Gel filtration	84,200	115,000	13	107
p.Tyr586Phe	Crude Extract	845,000	1650	100	1
	Affinity chromatography	287,000	15,800	34	10
	Gel filtration	128,000	109,000	15	65
P561X	Crude Extract	4,660,000	8990	100	1
	Affinity chromatography	3,110,000	156,000	66	17
	Gel Filtration	1,730,000	288,000	37	32
V562X	Crude Extract	6,410,000	13,300	100	1
	Affinity chromatography	2,960,000	116,000	46	9
	Gel filtration	1,330,000	402,000	21	30
H563X	Crude Extract	3,480,000	9070	100	1
	Affinity chromatography	2,990,000	170,000	86	19
	Gel Filtration	1,610,000	300,000	46	33
F575X	Crude Extract	1,510,000	2990	100	1
	Affinity chromatography	355,000	30,500	23	10
	Gel Filtration	174,000	134,000	12	45

^a^Data are averages from at least 3 independent purifications

**Fig. 1:**
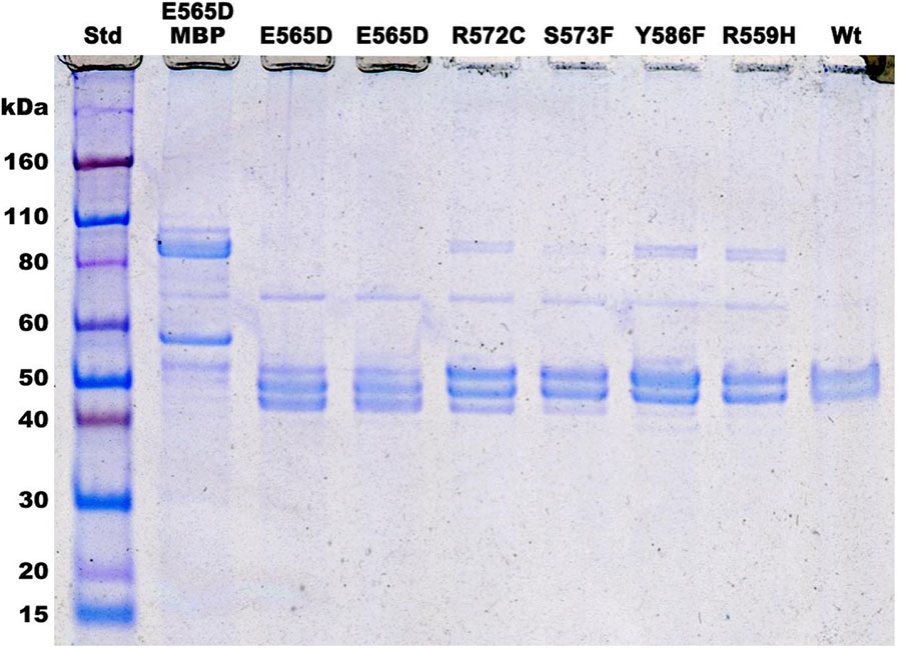
Protein standards (STD) are Novex Sharp Pre-stained. Lane 2 contains the E565D•MBP fusion protein following affinity purification while all remaining lanes are for enzyme after MBP removal and gel filtration chromatography by FPLC

**Fig. 2:**
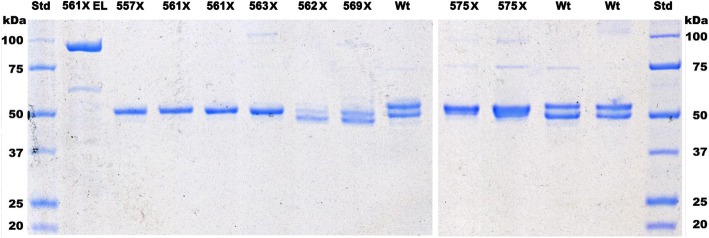
SDS Page profile of purified ALAS2 truncation (X) mutations. The 561X EL sample is prior to cleavage of the maltose binding protein moiety, while all others are after cleavage. Wt is wild-type ALAS2 and Std are Bio-Rad Precision Plus unstained protein standards. Two different gels were scaled linearly and aligned

The purification yields of the five missense mutations were all lower than that of the wild-type enzyme (23%), ranging from a very low 6% (p.R559H) to 19% of the initial crude extract activity. After gel filtration chromatography, the ALAS2 missense mutant preparations appeared nearly homogeneous on SDS-PAGE (Fig. 1). The purified wild-type recombinant enzyme existed in two forms of approximately equal amounts by densitometry; ~ 54 and ~ 52 kDa, with the lower form due to an ~ 2-kb carboxyl-terminal cleavage that occurred during expression and/or purification, despite the presence of protease inhibitors (Bishop et al. 2013). In addition, all of the missense preparations exhibited, to varying degrees, a third band of approximately 50 kDa. This band was prominent in the p.E565D and p.R572C preparations, but was present at much lower quantities in the p.R559H, p.S573F and p.Y586F proteins. It appeared to be a further degradation product of the upper two bands as they diminished in proportion to the appearance of the ~ 50 kDa band,

In contrast to the case of the missense mutations, the purification of three of the truncated enzymes (p.P561X, p.V562X, p.H563X), resulted in a higher yields (26–46%) than that of the wild-type enzyme (Table 1). The same result was found in our previous study for the p.Q548X and p.Q581X truncations (Bishop et al. 2013). The ALAS2 truncation mutants p.P561X and pH563X, after fusion-protein cleavage and FPLC purification, migrated to the 52 kDa region (Fig. 2), similar to the p.F557X mutant, which was previously shown to correspond in size to the lower band of wild-type ALAS2 (Ref (Bishop et al. 2013), see Fig. 2). On the other hand, truncation mutant p.V562X migrated further to a position corresponding to a size of around 50 kDa. The E569X mutant exhibited both the 50 and 52 kDa bands. The F575X protein migrated to a position slightly lower in size than that of the upper, wild-type 54 kDa bands.

### Kinetic and Thermostability properties of the wild-type and mutant ALAS2 enzymes

The specific activities of all the purified missense mutation enzymes were modestly increased from 1.06 to 1.30 times that of the wild-type enzyme. For the substrate succinyl-CoA, the purified mutant enzymes exhibited modest decreases in K_m_ (1.1 to 1.5-fold) consistent with modest increases in V_max_, (1.26 to 1.87 fold) (Table 2). The Hill n numbers were all similar to that of the wild type enzyme. The thermostabilities of the ALAS2 gain-of-function missense mutation enzymes were increased ~ 2- to 3-fold compared to that of the wild-type enzyme. Of interest, this increase was greater, the further the mutation was away from the carboxyl-terminus.

**Table 2:** Kinetic and thermostability vs predicted properties of ALAS2 missense mutants^a^

Parameters	Wild type	R559H	E565D	R572C	S573F	Y586F
Purified SA (U/mg)	93,500	100,000	122,000	121,000	115,000	109,000
Fold–increase	1.00	1.06	1. 30	1.29	1.23	1.17
V_max_						
Succinyl-CoA (U/mg) ± StdDev	93,500±5300	175,000±2190	143,000±7000	136,000±14,100	120,000±6710	146,000±10,100
Fold-increase	1.00	1.87	1.53	1.45	1.26	1.56
K_m_						
Succinyl-CoA (μM)	57.1 ± 6.7	46.3 ± 6.5	50.8 ± 2.15	46.7 ± 1.2	41.1 ± 9.2	44.0 ± 5.2
Fold-decrease	1.00	1.2	1.1	1.2	1.4	1.3
Hill coeff. (n)	1.6 ± 0.1	1.65 ± 0.08	1.5 ± 0.02	1.7 ± 0.17	1.45 ± 0.11	1.49 ± 0.14
T_1/2_ 45 °C (min)	4.7 ± 1.4	13.8 ± 3.4	13.6 ± 2.8	11.8 ± 1.5	9.3 ± 0.8	7.5 ± 0.2
Prediction Program (Ref) and Score^b^						
PolyPhen-2 (Adzhubei et al. 2010)		Benign	Benign	~Damaging	~Damaging	Benign
Score		0.003	0.001	1.000	0.995	0.016
SIFT (Kumar et al. 2009)		Tolerated	Tolerated	Deleterious	Deleterious	Tolerated
Score		0.561	0.335	0.000	0.001	0.101
Mutation Assessor (Reva et al. 2011)		Low	Neutral	Low	Medium	Neutral
Score		1.32	−0.285	1.94	2.12	0.46
Provean (Choi et al. 2012)		Neutral	Neutral	Deleterious	Deleterious	Neutral
Score		−0.45	−0.47	−3.28	−2.75	−0.89

^a^Data are means ±SD for *n* = 4–5 separate experiments and V_max_, K_m_, and T_1/2_ results all show statistically significant differences from the corresponding wild-type values

^b^Data are the values returned by the online prediction servers for each listed program

In contrast to the modest increases in specific activity accompanying missense mutations in the carboxyl-terminal region of ALAS2, truncation mutations in this region markedly increased specific activities from 1.4 to 4.0 fold (Table 3). This was mirrored in similar increases in V_max_ for both succinyl-CoA (1.7 to 5.6 fold) and glycine (1.6 to 4.0 fold over wild-type). However, there were only modest changes in the substrate affinities with the K_m_ values for substrate succinyl-CoA being mostly decreased (0.9 to 1.5 fold below wild-type) and the K_m_s for glycine were mostly increased (0.96 to 1.5-fold over wild-type).

**Table 3:** Kinetic and thermostability properties of ALAS2 truncation mutants^a^

Parameters	Wild-Type	P561X	V562X	H563X	E569X	H575X
Purified SA (U/mg)	81,200	288,000	322,000	300,000	143,000	134,000
Fold–increase	1.0	3.6	4.0	3.7	1.8	1.4
V_max_ Succ. CoA (U/mg) ±SD	103,000 ± 9700	351,000 ± 25,000	581,000 ± 88,600	316,000 ± 3940	182,000 ± 9570	163,000 ± 26,600
Fold-increase	1.0	3.4	5.6	3.1	1.8	1.7
K_m_ Succ. CoA (μM) ± SD	56.1 ± 6.7	43.3 ± 2.3	38.5 ± 2.0	39.6 ± 4.6	48.8 ± 5.8	51.2 ± 2.5
Fold-decrease	1.00	1.3	1.5	1.4	1.1	1.1
Hill coeff. SCoA (n)	1.6 ± 0.04	1.5 ± 0.1	1.6 ± 0.1	1.6 ± 0.1	1.8 ± 0.1	1.6 ± 0.1
V_max_ Gly (U/mg) ± SD	96,700 ± 21,400	324,000 ± 18,800	381,000 ± 40,000	316,000 ± 15,000	156,000 ± 15,700	153,000 ± 23,700
Fold-increase	1.0	3.4	4.0	3.3	1.6	1.6
K_m_ Gly (mM) ± StdDev	9.9 ± 0.5	13.1 ± 0.7	9.5 ± 2.2	14.4 ± 3.1	10.0 ± 2.0	10.4 ± 1.3
Fold-increase	1.0	1.3	0.96	1.5	1.0	1.1
T_1/2_ 45 C (min) ± StdDev	4.7 ± 0.6	2.5 ± 0.7	1.0 ± 0.1	3.3 ± 0.4	3.8 ± 0.3	ND^b^
Fold-decrease	1.0	1.9	4.7	1.4	1.2	

^a^Where provided, data are means ±SD for *n* ≥ 3 separate experiments

^b^ND = Not Determined

**Fig. 3:**
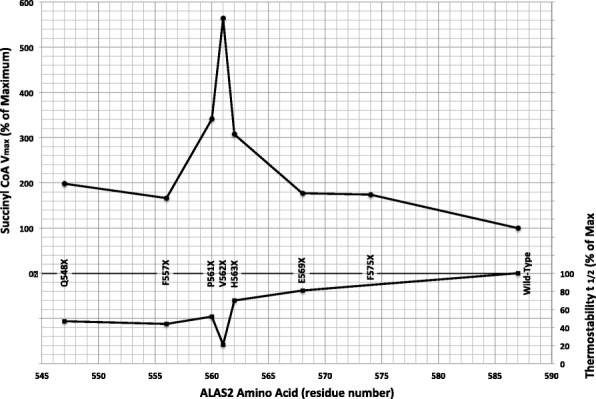
Effect of Truncation on Succinyl-CoA Vmax and Thermostability. The V_max_ and thermostability values are from Table 3

Notably, most of the thermostabilities were significantly reduced with half-lives at 45 °C of 1.2 to 4.7-fold decreased over wild-type. For both substrates, therefore, the major kinetic effect was due to greatly increased substrate turnover rather than increases in substrate affinity. Furthermore, unlike the missense mutations, the truncation mutations resulted in enzymes that were more thermolabile than wild-type with half-lives up to 5 times shorter. Interestingly, the most thermolabile mutant had the greatest increases in enzyme rate compared to the wild-type enzyme (Fig. 3).

## Discussion

In our previous publications (Bishop et al. 2012; Bishop et al. 2013) we investigated the biochemical bases of the *ALAS2* exon 11 carboxyl-terminal mutations that cause XLP and XLSA. These results demonstrated that some specific mutations could cause loss-of-function and thus XLSA, along with loss of succinyl-CoA synthase binding, while others resulted in gain-of-function by releasing intrinsic inhibition of ALAS2 by its carboxyl-terminus, thus causing XLP. In this study, we examined the kinetic and thermostability properties of additional selected missense and truncation mutations throughout the ALAS2 carboxyl-terminal region in order to further define the structural and kinetic parameters influencing the regulatory properties of ALAS2 exon 11.

The polymorphisms selected for this study constituted all the missense mutations in exon 11 that had been identified by the ESP database. There were five exon 11 mutations in the ESP database. (p.R559H, p.E565D, p.R572C, p.S573F, and p.Y586F) with allele frequencies of 0.009 to 0.294% (Additional file 2: Table S2). More recently, the spectrum of polymorphisms in this region has been increased and published in the Exome Aggregation Consortium (ExAC) database (Lek et al. 2016). This project achieved a nearly 10-fold increase in sampling from 6503 individuals (ESP) to 60,706 individuals (ExAC). There were eight additional exon 11 missense mutations found in the ExAC database and the most recent Genome Aggregation Database (gnomAD) (The genome Aggregation Database (gnomAD) 2018) (see Additional file 2: Table S2), but all the additional variants had low allele frequencies of 0.002 to 0.006%, corresponding to 1 to 3 individuals with each mutation. Thus, the survey of the ESP variants represents the vast majority of individuals expected to exhibit possible alteration of function mutations in ALAS2 exon 11.

The most frequent SNP (p.R559H) was previously identified in an 80 year-old female with late-onset XLSA (Pereira et al. 2004). To date, the present is the only report of this variant’s expression, purification and kinetics. The purified mutant enzyme had essentially the same specific activity as the wild-type enzyme. Kinetically, it had a modestly lower K_m_ and higher V_max_ for succinyl-CoA (Table 2). However, the purification yield was only 9% — 60% lower than that of the wild-type enzyme. This low yield is likely due to proteolysis during purification rather than thermolability since it’s half-life at 45 °C was nearly 3-fold greater than that of the wild-type enzyme (Table 2). Since this mild mutation eventually resulted in sideroblastic anemia in an elderly female, these results suggest that the enzyme is more susceptible than wild-type to proteolytic degradation in vivo. Alternatively, it is noted that this mutation is within the region previously suggested to be a site for interaction with succinyl CoA Synthase (Bishop et al. 2012). In those cases, as here, the mutation has little effect on ALAS2 catalytic activity, but results in sideroblastic anemia in vivo (Kadirvel et al. 2012).

To date, there have been no reports of disease associated with the polymorphisms p.E565D, p. R572C or p.S573F. Here, we report that all three SNPs had slightly increased specific activities (1.2- to 1.3-fold), modestly lower K_m_s and slightly higher V_max_ values (1.3- to 1.5-fold) for succinyl-CoA and 2- to 3-fold greater thermostabilities (Table 2). These properties would suggest modestly increased ALA synthesis in vivo, but possibly not sufficient to cause XLP.

The last missense mutation (p.Y586F) has been previously implicated as an enabler of congenital erythropoietic porphyria (CEP) (To-Figueras, J 2011). In that instance, phenotypic heterogeneity with respect to porphyrin accumulation was discovered in four unrelated CEP patients, all with the same compound heterozygous uroporphyrinogen synthase (UROS) p.C73R/p.P248Q genotype. While three patients had mild disease, one was more severe, with about five-fold higher urinary porphyins than the others. She alone also had an ALAS2 p.Y586F mutation that resulted in about 1.15-fold higher specific activity compared to wild type enzyme. Here, we found that purified p.Y586F enzyme had a V_max_ for succinyl CoA that was 1.6-fold higher than wild type. Thus, this gain-of-function carboxyl-terminal polymorphism can increase uroporphyrin accumulation in a CEP patient who already had underlying heterozygous UROS mutations (To-Figueras, J 2011).

It was notable that the increase in SNP specific activity was greater, the further the mutation was away from the carboxyl-terminus. Thus, the native conformation of the carboxyl-terminal region may be destabilizing to the enzyme structure, with various point mutations in part relieving this destabilization to a greater extent, the closer the mutation is to residue R559.

The unique role of the ALAS2 carboxyterminal region in the regulation of ALAS2 activity was reflected in the general failure of in silico phenotype prediction programs to correlate with the in vitro expression results reported here. These results are summarized in Table 2 where there is little correlation between predictions and the universal low to modest increase in V_max_ for succinyl CoA. This might be expected, since these programs are designed to detect substitutions that have a major deleterious effect locally, where the ALAS2 carboxyl terminal region appears to be more heterogeneous in mechanism with similar amino acid substitutions able to dramatically influence enzyme conformation, typically leading to gain of function, while in other cases, possibly for R559H, to alter an enzyme binding site region (Bishop et al. 2012).

In summary, it appears that the five common SNPs in the ALAS2 carboxyl-terminal region presumably have clinical significance limited to their role as modifying genes – that only in concert with other underlying disturbances of heme biosynthesis may increase ALA synthesis and porphyrin accumulation sufficiently to result in phenotypic disease. These SNPs also did not have a marked effect on ALAS2 kinetics and thus seem more tolerated in the carboxyterminus than mutations elsewhere in the enzyme. Nonetheless, these SNPs should be considered as possible modifier gene mutations that could enhance the symptoms of a porphyria.

In contrast, truncation mutations introduced in this region resulted in high levels of ALAS2 activity typical of mutations causing XLP. This is analogous to most known XLP mutations that cause frameshifts, thereby effectively truncating the ALAS2 C-terminus (Whatley et al. 2008; Bishop et al. 2013). Four of the five new truncation mutations had markedly increased rates of substrate consumption; 1.8- to 5.6-fold for succinyl-CoA and 1.6- to 4.0-fold for glycine (Table 3).

Similar to results for expressed XLP mutations (Bishop et al. 2013), the p.P561X, p.V562X, and p.H563X truncation mutants showing 1.4- to 4.7-fold reduced half-lives at 45 °C (Table 3). Of interest, when these data were compared to the succinyl-CoA V_max_ values, the basic trend was an inverse relationship (Fig. 3). This could indicate that gain of function in this region is due to increased flexibility or structural “openness” that results in both thermolability and more rapid substrate turnover. In support of this hypothesis, Fratz, et al., have confirmed that the p.Q548X and ΔAGTG mutations that cause XLP have significantly reduced thermostabilities and altered active site structural environments by near-UV circular dichroism spectroscopy, consistent with conformational changes that enhance the rate of succinyl-CoA utilization and product (ALA) release – possibly mediated through iron or heme binding to the Cys-X-X-Cys motif located at residues 555 to 558 (Fratz et al. 2015), by interactions with succinyl-CoA synthase near residues 567 and 568 (Bishop et al. 2012) or by the predicted α-helical structure of residues 564 to 574 (Bishop et al. 2013). It is therefore important to consider the reciprocal relationship of thermostability and catalytic activity for truncation mutations in the gain-of-function region (Fig. 3). The region of maximal enzyme activity and minimal thermostability coincides with truncations at residues 561 to 563. Since these truncations are after the Cys-X-X-Cys motif, but before the putative alpha helix, it is more likely that the latter is the key regulatory motif. Thus, the alpha helical region may normally stabilize the protein in a manner that reduces active site conformational flexibility and down-regulates ALA product release.

The variations in subunit sizes of the mutated ALAS2 polypeptides as assessed by denaturing SDS PAGE are of interest. The wild-type enzyme and the SNPs all resulted in two similar forms with apparent molecular weights on SDS gels of around 54 and 52 kDa. The upper band corresponds to the full-length peptide from amino acids aspartate 79 to alanine 587 with a calculated molecular weight of 56.4 kDa. The lower band, is equivalent to the slightly shorter Asp79 to Asn556 polypeptide (Bishop et al. 2012; Bishop et al. 2013) with a calculated molecular weight of 52.6 KDa. The missing 31-amino acid polypeptide has a calculated molecular weight of 3.8 kDa. Thus, the apparent molecular weights on SDS gels are about 1–2 kDa less than the actual molecular weights. It should also be noted that mass spectrometry identified Ala88 as the most amino-terminal amino acid (Bishop et al. 2012). This may simply be due to a difficulty in purifying or recovering the first peptide, but if the prokaryotically expressed and purified ALAS2 initiates at Ala88, its predicted 51.6 kDa size is significantly smaller than the observed size in gels.

In contrast to the two molecular sizes observed for the SNPs, the truncation mutations tend to result in only the lower ~ 52 kDa band. This lower band corresponds to an enzyme form with higher activity than that of the upper band (Table 2, mutant p.F557X, reference #10), confirming that the native C-terminal residues function as a down-regulator of ALAS2 enzymatic activity. Since the dramatic increase in ALAS2 V_max_ for succinyl-CoA was not accompanied by a corresponding major increase in substrate affinity (only a modest decrease in K_m_ was observed), and since the stabilities of these enzymes were decreased with increasing extent of truncation, it remains that the increased turnover is most likely due to enhanced product release due to the loss of the C-terminal residues.

The presence of the putative alpha helix at residues 564–574 (Bishop et al. 2013) appears to be important, as we show that the maximum gain-of-function occurred for truncations of any of the three residues just prior to the alpha helix region. It appears that contact of the helix with the ALAS2 protein may increase its rigidity and/or compactness, protecting it from proteolysis and reducing its conformational flexibility. Conversely, when truncated, the increased flexibility may result in easier product release, and the consequent major increase in activity. Previously, Lendrihas, Hunter and Ferreira (Lendrihas et al. 2010; Hunter and Ferreira 2011) showed that conformational flexibility was critical for ALA release from ALAS2. In their study, mutations in an active site-resident loop in the murine enzyme corresponding to human exon 10 residues 500 to 517 were proposed to alter ALA release rate via conformational change of the loop. The shortened half-lives for thermal denaturation of the ALAS2 truncation mutations was consistent with an increase in instability and flexibility of the protein that could result in the observed increased in enzymatic activity due to increased rates of ALA release. Stojanovski, et al., has also recently suggested that truncation mutations that change the ALAS2 tertiary structure from a closed to open conformation may increase activity by releasing product more rapidly and may decrease thermostability by reducing the rigidity of the structure (Stojanovski et al. 2016).

Of note, while this manuscript was in review, the structure of erythroid-specific ALAS2 (PDB ID 6HRH) was deposited in the RCSB PDB Database (Bailey et al. 2018). Viewing the deposited structure with the MacPyMol molecular visualizer (The PyMOL Molecular Graphics System 2018) shows that the region around F557 is unstructured and does not appear in the deposited crystal structure. This supports our findings that mutations and deletions in the carboxyl terminus distal to this region could result in the enzyme being more susceptible to protease activity. Most importantly, our viewing the stereo image of the deposited structure reveals that the above-described alpha helical structure consisting of residues S566 to F573, lies on the surface of each subunit directly above the active site loop reported by Lendrihas, (Lendrihas et al. 2010) as well as above the PLP active site moiety. Thus it is clear that this helix could inhibit ALA release as well as promoting enzyme rigidity, thus providing a rationale as to why the maximum deletion mutant activation centers on this helix (see Fig. 3).

The regulatory nature of the ALAS2 carboxyl-terminus has been shown here to significantly involve an alpha helix near the center of a eukaryotic-specific sequence extension of the core ALAS sequence found in prokaryotes. The mitochondrial foldase-protease complex, ClpXP has also been shown to play a significant role in ALAS2 regulation in that ClpX unfoldase activates ALAS2 by stimulating incorporation of cofactor pyridoxal 5′-phosphate (PLP) into ALAS2 (Kardon et al. 2015) and that a mutation in ClpX modulates ALAS2 levels in humans (Yien et al. 2017). Since the new crystal structure of ALAS2 shows that there is a cone-shaped access hole to PLP that is near the carboxyl-terminus, it would be interesting to know if this region is involved with ClpXP. Importantly, Kubota, et al. showed that the Heme Regulatory Motif, amino acids CPF/L (Lathrop and Timko 1993), present in mitochondrial ALAS2 mediated heme-dependent recruitment of ClpPX for degradation of ALAS2 (Kubota et al. 2016). Since there is a surface-localized CPL sequence at the cone-shaped entrance to the PLP binding site near the carboxyterminus, the involvement of the eukaryotic-specific carboxyterminus extension may be of importance to multiple aspects of the regulation and control of ALAS2 activity.

## Conclusions

An ALAS2 C-terminal SNP (p.R559H) had markedly low purification yield suggesting enzyme proteolytic instability as a possible cause for the previously identified late-onset patient with XLSA.

Four relatively common SNPs (p.E565D, p.R572C, p.S573F, and p.Y586F) in the exon 11 C-terminal region were associated with small increases in enzymatic activity in vitro, and are likely biochemical and clinical modifiers of porphyria severity.

Synthetic ALAS2 exon 11 truncation mutations centered on an active-site-covering alpha helix resulted in dramatic increases in succinyl-CoA turnover that were largely independent of changes in substrate affinity, thus correlating increased thermolability and presumably increased conformational flexibility with enhanced ALA product release.

## Additional files

Additional file 1: **Table S1.** Oligonucleotides primers (5′ to 3 ‘forward) for mutagenesis and sequencing of ALAS2. (DOC 36 kb)

Additional file 2: **Table S2.** Allele frequencies of *ALAS2* exon 11 SNPs in the ESP, EXAC and GnomAD databases. (DOC 25 kb)

## Abbreviations

ALA: 5-Aminolevulinic acid
ALAS2: 5-Aminolevulinic acid synthase, erythroid isozyme
CEP: Congenital erythropoietic porphyria
CoA: Coenzyme A
ESP: Exome Sequencing Project
ExAC: Exome Aggregation Consortium
K_m_: Michaelis kinetic constant
SDS-PAGE: Sodium dodecyl sulfate – polyacrylamide gel electrophoresis
SNP: Single nucleotide polymorphism
UROS: Uroporphyrinogen synthase
V_max_: Maximum reaction velocity at saturating substrate
XLP: X-linked protoporphyria
XLSA: X-linked sideroblastic anemia
