# Supplementary material for: Molecular expression, characterization and mechanism of ALAS2 gain-of-function mutants
Source: Mol Med. 2019 Jan 24;25:4. doi: 10.1186/s10020-019-0070-9 (PMC6344999; doi:10.1186/s10020-019-0070-9)
Supplement: Supplementary file 1 — Table S1. Oligonucleotides primers (5′ to 3 ‘forward) for mutagenesis and sequencing of ALAS2. (DOC 36 kb) [file 10020_2019_70_MOESM1_ESM.doc]

SUPPLEMENTARY DATA

Table S1. Oligonucleotides primers (5’ to 3 ’forward*) for mutagenesis and sequencing of ALAS2

Mutations^a^ Sequence^b^ ALAS2 Location

R559H 5’-TGCCTGCAATTTCTGTCACCGTCCTGTACACTTTG-3’ 1676 G>A

E565D 5’ -GTCCTGTACACTTTGACCTCATGAGTGAGTGGG -3’ 1695 G>C

R572C 5’-TCATGAGTGAGTGGGAATGTTCCTACTTCGGGAAC-3’ 1714 C>T

S573F 5’ –GAGTGAGTGGGAACGTTTCTACTTCGGGAACATGG-3’ 1718 C>T

Y586F 5’ -GGCCCCAGTATGTCACCACCTTTGCCTGAAATTC -3’ 1757 A>T

P561X 5-‘GCCTGCAATTTCTGTCGCCGTTAGGTACACTTTGAGCTCATGAGTG-3, 1681-3 CCT>TAG

V562X 5’-GCAATTTCTGTCGCCGTCCTTAATAGTTTGAGCTCATGAGTG-3’ 1684-6 GTA>TAA

H563X 5’ -CTGTCGCCGTCCTGTATAGTTTGAGCTCATGAGTGAGTGGG -3’ 1687-9 CAC>TAG

E569X 5’ –GTACACTTTGAGCTCATGAGTTAGTGGGAACGTTCCTAC -3’ 1705-7 GAG>TAG

F575X 5’ –GGAACGTTCCTACTAGGGGAACATGGGC -3’ 1723-5 TTC>TAG

Oligo 1F 5’-TGTTGC CAATGACTCTACTC -3’ 828-848

Oligo 2R 5’- GTGCCAAGCTTGCCTGCA -3’ (pMALc-2 –AE2) 4419- 4402

Oligo 3F 5’ - CGCAATGTCAAGCACATGCG -3’ 1354-1373

a. Reverse mutant primers are not shown, but correspond to the reverse complements of Forward primers.

b. The underlined G residue corresponds to the mutation of codon 588 from TGA to TGC so that there will only be one stop codon in the transcript.
