# Supplementary material for: Molecular expression, characterization and mechanism of ALAS2 gain-of-function mutants
Source: Mol Med. 2019 Jan 24;25:4. doi: 10.1186/s10020-019-0070-9 (PMC6344999; doi:10.1186/s10020-019-0070-9)
Supplement: Supplementary file 2 — Table S2. Allele frequencies of ALAS2 exon 11 SNPs in the ESP, EXAC and GnomAD databases. (DOC 25 kb) [file 10020_2019_70_MOESM2_ESM.doc]

SUPPLEMENTARY DATA

Table S2. Allele frequencies of *ALAS2* exon 11 SNPs in the ESP, EXAC and GnomAD databases.

**SNP Coordinate Base Amino Acid ESP EXAC GnomAD**

(rs#) (chromosome) (change) (change) (frequency^a^) (frequency^b^) (frequency^b^).

rs763862539 X:55035756 T > A  T541S — — 0.0002

— X:55035746 C > A G544V — — 0.0006

— X:55035738 G > C L547V — — 0.0006

rs773629116 X:55035733 C > A  Q548H — — 0.0047

rs777143293 X:55035723 C > G  V552L — — 0.0006

rs145704441 X:55035701 C > T  R559H 0.2940 0.3320 0.2137

rs778388971 X:55035699 G > A  R560C — — 0.0043

rs149747514 X:55035682 C > G  E565D 0.0470 — —

rs372280573 X:55035663 G > A  R572C 0.0100 0.0020 —

rs201799139 X:55035659 G > A  S573F 0.0100 0.0340 0.0280

rs754879867 X:55035651 C > T  G576R — — 0.0047

rs766555892 X:55035645 T > C  M578V — — 0.0006

rs766555892 X:55035645 T > G  M578L — — 0.0024

rs139596860 X:55035620 T > A  Y586F 0.0260 0.1130 0.0683

a. Frequency is for a European population.

b. Frequency is for all populations
